# Achenbach Syndrome: An Atypical Presentation on the Left Hand

**DOI:** 10.7759/cureus.81004

**Published:** 2025-03-22

**Authors:** Aurelia Incristi, Justin Lindsay, Murad Nawaz, Ryan O' Donnell, Stephanie Johnson

**Affiliations:** 1 Medicine, Wright State University Boonshoft School of Medicine, Dayton, USA; 2 Pathology and Laboratory Medicine, Wright State University Boonshoft School of Medicine, Dayton, USA

**Keywords:** achenbach syndrome, blue finger, paroxysmal finger haematoma, spontaneous hematoma, upper extremity vascular disorder, vascular medicine

## Abstract

Achenbach syndrome is a benign condition of unknown etiology characterized by sudden onset bluish discoloration and bruising of one or more fingers without prior trauma, typically affecting the proximal phalanges on the dominant hand while sparing the nail bed and distal phalanges. It predominates in women, most often in their fifth decade of life, and is diagnosed clinically. This case presents a 59-year-old female patient with spontaneous painful pruritic bruising of her left middle finger, which spared the nail bed but not the fingertip. This case highlights an atypical presentation on the non-dominant hand involving the distal phalanx, which is typically spared. The course of the presentation lasted four days, at which point symptoms resolved. The patient reported taking aspirin for pain following the onset of symptoms with no improvement and denied previous occurrences of symptoms. Based on clinical presentation and symptoms, the patient was diagnosed with Achenbach syndrome. When the patient discovered Achenbach syndrome was benign, they denied consent for any diagnostic testing or imaging. The case aims to increase awareness and understanding of clinical variations of this condition by illustrating an unusual presentation on the non-dominant hand involving the distal phalanx so clinicians can ease patient anxiety and patients are not subjected to unnecessary testing or potential misdiagnosis.

## Introduction

Achenbach syndrome, also known as paroxysmal finger hematoma, is a condition of unknown etiology first described in 1958 by Walter Achenbach [1-5]. Since then, an estimate of fewer than 100 cases has been reported in the literature [2,4]. Achenbach syndrome is characterized by sudden onset bluish discoloration and bruising of one or more fingers without prior trauma [1-4]. Typically, the proximal phalanges on the dominant hand are affected; the nail bed and distal part are spared [2,4]. Symptoms of pain, swelling, and itching are commonly reported, and patients mainly experience discoloration on the palmar side of the finger [1-4,6]. The disease is benign and resolves within several days without treatment [1,5,6]. While the etiology is unknown, various proposed mechanisms include subcutaneous bruising, venous hemorrhaging, and increased vascular fragility [1,4,5,7,8]. It predominates in women, most often in their fifth decade of life, and relapses are common [1,2,5,6]. Diagnosis is clinical due to the majority of routine physical examinations and blood screens showing normal results [4].

We present a 59-year-old woman with symptoms supporting a diagnosis of Achenbach syndrome with the goal of increasing awareness of this condition. Clinical photographs taken throughout the disease presentation were collected and documented to allow for comparison to previous cases and demonstrate the variation in the clinical presentation of Achenbach syndrome.

## Case presentation

**Figure 1:**
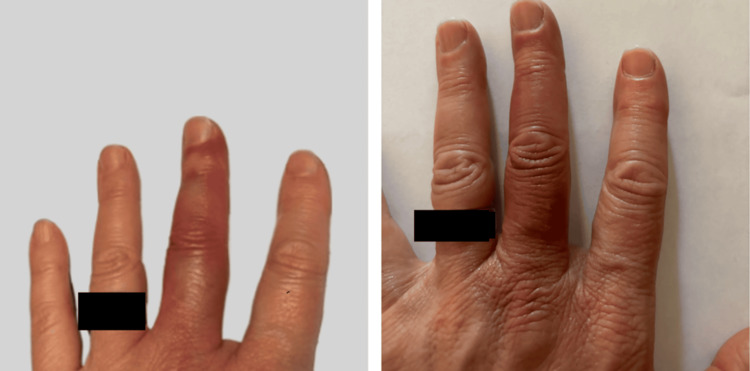
Achenbach Syndrome Presentation on Non-dominant Hand A 59-year-old woman with spontaneous painful pruritic bruising of her left (non-dominant) middle finger that spared the nail bed but not the distal phalanx.

A 59-year-old female patient noticed spontaneous ecchymosis of her left (non-dominant) middle finger accompanied by burning pain and itching (Figure 1). The bruising initially started on the proximal palmar portion of the left middle finger and then progressed circumferentially over several hours to involve the entirety of the finger, sparing the fingernail. As the ecchymosis progressed to involve the entire finger, the bluish discoloration darkened to include purple hues. The course of the presentation lasted four days, at which point symptoms resolved. The patient reported taking aspirin and icing the finger following the onset of pain and discoloration, with no improvement or worsening of symptoms from either treatment. She denied any previous occurrences of symptoms and has no history of amyloid disease, clotting disorders, vitamin C deficiency, thrombocytopenia, Raynaud’s phenomenon, or any familial genetic conditions. The patient reported a pharmaceutical history that included Enalapril 10 mg by mouth daily for well-managed hypertension, but this was not considered as contributing to her condition, as no medications have been associated with Achenbach syndrome [5]. The patient denied smoking history, chemical, or dye exposure and did not undergo any laboratory testing. Based on clinical presentation, the patient was diagnosed with Achenbach syndrome with an atypical presentation involving the distal phalanx on the non-dominant hand. The patient denied consent for any diagnostic testing or imaging due to the benign nature of the condition, so the diagnosis was made based on symptom presentation and past medical history. Due to spontaneous symptom resolution, no treatment was administered.

## Discussion

Achenbach syndrome is a benign condition of unknown etiology that presents with unilateral bruising, pain, and swelling involving the palmar aspect of the middle, ring, or index finger [1,2,4]. Traditionally, the disease involves the proximal index or middle finger on the dominant hand [2,4]. Achenbach syndrome is diagnosed clinically due to normal findings upon physical exam and diagnostic screenings, such as Allen's test, Doppler ultrasound, angiography, capillaroscopy, coagulation studies, immunological and biochemical profiles, complete blood count, and ankle-brachial index [1,4]. Prodromal symptoms such as pain, tingling, and itching can last either a few minutes or multiple hours [1,4]. The condition is self-resolving and can disappear after local pressure is applied [4,7].

Various underlying mechanisms have been proposed, one of which includes subcutaneous bleeding involving venous rather than arterial hemorrhaging [4,7]. The violaceous discoloration seen in Achenbach syndrome resembles subcutaneous bruising, but patients do not experience the traditional color changes of a healing ecchymosis (green, yellow), and resolution is spontaneous rather than fading over several weeks [7]. Another proposed mechanism of Achenbach syndrome involves increased vascular fragility and capillary resistance, which may cause microhemorrhages that occur with or without minor trauma [1,2,5,8]. Additionally, punch skin biopsies conducted on a small number of patients with Achenbach syndrome suggest evidence of multiple thin-walled ectatic vessels in the dermis with extravasation of erythrocytes as well as hyperkeratosis and parakeratosis of the epidermis [1,8]. Vascular permeability and extravasation of erythrocytes can be altered through a complex interplay of inflammatory mediators (bradykinin, histamine, vascular endothelial growth factor) binding to receptors expressed on endothelial cells, thereby opening the junctional barrier and disrupting the organization of the interendothelial junction that mediates paracellular transport [9]. While these mediators have not been studied in Achenbach syndrome patients, it is possible that these general mechanisms of vascular permeability may contribute to the extravasation of erythrocytes seen in the dermis of Achenbach patients, which could account for the associated color changes and pain seen in Achenbach syndrome [1,8,9]. Finally, it has been hypothesized that Achenbach syndrome could be due to vasospasm from reduced blood flow [2,8]. There has been no evidence to suggest thromboembolic events as a potential cause of this disease [1,5].

In terms of clinical presentation, a previous analysis of 24 patients showed that Achenbach syndrome most commonly presents in the right-hand (dominant) index finger, while this study demonstrates a clinical variation involving discoloration in the left-hand (non-dominant) middle finger [8]. Our unusual presentation illustrates the variability of this disease and allows for further characterization of clinical features. It is imperative that the medical community be able to distinguish this benign self-resolving condition from other more serious conditions that involve discolorations of the digits, including peripheral vascular disease or embolic phenomena [3].

Furthermore, potential differential diagnoses that should be considered include Raynaud’s syndrome, acrocyanosis, acute limb ischemia, spontaneous digital venous thrombosis, microemboli, collagen vascular disease, and Gardner-Diamond syndrome [2-5,8]. However, Raynaud's syndrome is associated with cold temperatures and is chronic rather than episodic, as seen in Achenbach syndrome [3,4]. Acrocyanosis presents symmetrically, rather than unilaterally, and is common in children and young adults of both genders, rather than middle-aged females [3]. Acute limb ischemia, while sudden in onset, differs from Achenbach syndrome in that there is no female predominance, normal peripheral temperature, and often no palpable pulses in the affected extremity [2,3]. Achenbach syndrome is not characterized by increased fibrin deposition, eliminating a thrombotic source [1]. Gardner-Diamond syndrome, while displaying a female predominance, can affect any part of the body and is associated with personality disorders, syncope, bleeding, and joint pain [3,4]. Further understanding of this condition can help physicians ease patient anxiety and assure them that their condition is benign and self-resolving.

## Conclusions

Achenbach syndrome is an uncommon benign condition with an etiology that is largely unknown. While previous studies have shown a predominant presentation in the dominant index finger sparing the distal phalanx, this case study illustrates a variation in clinical presentation involving unusual discoloration of the middle finger on the non-dominant hand extending to the distal phalanx. While there is no specific treatment for this condition, it can cause significant patient anxiety, and patients could be subjected to unnecessary diagnostic testing, such as coagulation studies, ankle-brachial index testing, vascular imaging, and capillaroscopy. By increasing awareness and demonstrating the clinical variability of this condition, physicians can alleviate patient anxiety, limit excessive diagnostic testing, and prevent potential misdiagnosis.
